# The Impact of the COVID-19 Pandemic on Family Medicine Practices in Saudi Arabia

**DOI:** 10.7759/cureus.20437

**Published:** 2021-12-15

**Authors:** Mokhtar Shatla, Bassam T Alharthi, Abdullah G Alharbi, Zakaria A Khan, Anwar A Althaqfi, Abdulrahman A Babkoor, Ahmed A Almalki

**Affiliations:** 1 Family and Community Medicine, Faculty of Medicine, Umm Al-Qura University, Makkah, SAU; 2 Otolaryngology, Faculty of Medicine, Umm Al-Qura University, Makkah, SAU; 3 Family Medicine, Faculty of Medicine, Umm Al-Qura University, Makkah, SAU; 4 Medicine, Faculty of Medicine, Umm Al-Qura University, Makkah, SAU; 5 Medcine, Faculty of Medicine, Umm Al-Qura University, Makkah, SAU

**Keywords:** health care worker, practice, knowledge, saudi arabia, covid19, family medicine

## Abstract

Background

The COVID-19 pandemic is one of the largest global healthcare crises in nearly a century. To face this global health emergency, health institutions have had to readjust their functioning while ensuring the continuity of care and protecting medical staff and patients. Our aim in this study was to assess the consequences of the COVID-19 outbreak on family medicine and its practice in Saudi Arabia.

Methods

This cross-sectional study was conducted during the period from June 30, 2020, to July 20, 2020, by posting an online survey on social media platforms (WhatsApp and Twitter) and emailing physicians individually to collect data on the impact of the COVID-19 pandemic on family medicine and primary care practices during the period of lockdown in Saudi Arabia.

Results

A total of 382 primary healthcare (PHC) providers participated in the study (males: 213 (55.8%); females: 169 (44.2%)). The mean age and standard deviation of the population were 38.27±7.46. Most participants were from governmental health sectors. Participants revealed that they have a good level of knowledge regarding COVID-19. However, they indicated confusion regarding the knowledge due to changing recommendations or multiple-source information. Only 57.3% of respondents received relevant training on the use of personal protective equipment (PPE). The most frequently used PPE were surgical masks (100%) and gloves (98.4%). The highly protective N95 masks were used by only 55.7%. Many health care workers indicated a high rate of stress and anxiety about the COVID-19 pandemic. Data obtained are suggestive that there was a reduction in outpatient visits and a reduction in consultation time. Canceled physical examinations of the patients during the consultation were encountered most of the time. There was no shortage of medications, nasopharyngeal swabs, or sanitizers. However, an occasional lack of PPEs occurred. 64.4% of the respondents used online consultations with their patients. A shortage of health care workers during the pandemic in family medicine clinics was encountered by 63.3% of the participants.

Conclusion

Family medicine practices are adversely affected by pandemics and lockdowns following them. It has been reported that COVID-19 interferes with preventive, chronic, and acute care visits and increases mental health visits. Outpatient visits have also decreased as well as the amount of time spent in consultations. In addition, the transition from in-person clinics to telemedicine has happened. Perhaps these changes will delay the diagnosis and prescription refills.

## Introduction

The COVID-19 pandemic is one of the most significant global healthcare crises in nearly a century. It started in Wuhan, China, in late 2019 and spread worldwide, with nearly three million cases and over 200,000 deaths, which have consumed the capacity and resources of international healthcare systems [1].

To combat this, many forms of guidance have been provided to assist in risk mitigation of viral spread, potential therapy options to treat COVID-19 patients, and reorganization strategies for hospital departments to help manage the increased patient load [2,3].

To face this global health emergency that has overwhelmed the health systems worldwide, health institutions have had to readjust their functioning to cope with COVID 19 while ensuring the continuity of care and protecting medical staff and patients. As COVID-19 is here to stay in the world, we will have dynamic challenges in our healthcare system [4,5].

However, a bigger group of doctors who encounter COVID-19 cases has received little attention: those who provide primary care, such as family physicians, general practitioners (GPs), and pediatricians, most of whom work exclusively in primary care [6].

The demand for medical care in many countries has exceeded the available resources, leading to a reprioritization of the medical landscape. Chronic and non-urgent care in hospitals has been largely suspended to increase emergency and respiratory care capacity. Non-urgent and elective surgical procedures have seen a disruption due to the COVID-19 pandemic as well [7,8]. Due to the PPE shortage, many clinics have adapted new safety protocols to continue providing care to patients, including social distancing measures, wearing masks, and offering telemedicine to patients who do not need to be present in the clinic [9].

Primary health care (PHC) provides a strong foundation for the global response by reducing the risk of transmission, monitoring mild cases in home isolation, reducing the demand for hospital services, and ensuring access to healthcare. Thus, PHC can play a crucial role in cutting down on the pandemic's effects by maintaining and extending other health care services [10].

This COVID-19 pandemic and the preventive measures were challenging for PHC systems. Population lockdown led to delayed treatment for non-COVID-19 conditions, as reported in Canadian experience, which reported some changes in service delivery where almost all of them are delivered virtually. On average, there was one in-person patient appointment per week compared to 50-60 patient appointments per week in the period before the COVID-19 pandemic. Providers also reported witnessing people with mental health concerns, seeing fewer sick children, and postponing routine preventative care. Prenatal and newborn visits were being done most of the time, virtually. While in the United States, doctors illustrated other difficulties as changes in practice have come quickly from authorities without prior preparation or even clear guidance [11,12].

Saudi Arabia has taken several restrictive measures to prevent the spread of and reduce exposure to COVID-19 even before reporting any cases to minimize the chance of virus introduction into the country. These measures are summarized in the travel ban to China on February 6, 2020, and the ban on travelers from COVID-19 affected countries [13]. In addition, international Umrah pilgrims were prohibited from entering Makkah, in addition to the Prophet's Mosque in Madinah being closed [14]. On March 2, 2020, Saudi Arabia reported the first confirmed case of COVID-19 [15]. Later that month, further measures were taken, including closing schools and shifting to online virtual learning, suspending all domestic air travel, and suspending all sports activities. In April 2020, Saudi Arabia imposed a nationwide lockdown and started mass and extensive testing in communities [16].

There were limited studies to assess the impact of the COVID-19 outbreak on family medicine and its practice in Saudi Arabia. Hence, this study aims to assess the impact of the COVID-19 pandemic on family medicine and its practice in Saudi Arabia.

## Materials and methods

This cross-sectional study was conducted during the period from June 30, 2020, to July 20, 2020, by posting an online survey on social media platforms (WhatsApp and Twitter) and emailing physicians individually to collect data on the impact of the COVID-19 pandemic on family medicine and primary care practices during the period of lockdown in Saudi Arabia. Snowball sampling was facilitated by making the WhatsApp and Twitter posts shareable. Informed consent was obtained from participants by informing them about the purpose and benefits of the study. The questionnaire is presented in the Appendix and consists of multiple-choice questions with single or multiple answers that apply. All the questions were mandatory. 

The questionnaire comprised four sections. Section one included the demographic characteristics of the participants. Section two assessed the practitioners’ knowledge of the COVID-19 pandemic, e.g., source of knowledge, institutional training on COVID-19 recommendations, methods of protection of themselves and patients, training on using personal protective equipment (PPE), the rating of their knowledge of COVID-19, etc. Section three assessed the impact of COVID-19 on family medicine and primary care practices, e.g., change in the rate and timing of daily consultations, disruption of patients’ physical examinations and communication, interruption of management of acute or chronic diseases, change in the rate of mental consultations, delayed consultations with other specialties, etc. Section four included questions to assess workplace changes, e.g., availability of working staff, shortage of resources, re-arrangement of the workplace, including physical distancing, and triage, etc.

The study was approved by the Ethics and Research Review Committee of Umm Al-Qura University, Faculty of Medicine (Approval number: HAPO-02-K-012-2021-03-601), and the online questionnaire was pilot tested with 50 participants. The results of the pilot test were included in the final results of the study [17].

Statistical analysis

Data were analyzed with STATA (StataCorp LLC, College Station, TX, USA). We represented categorical variables in terms of frequency (percentage). The binary logistic regression model was used to identify factors associated with mental changes. Odds ratios and their associated 95% confidence intervals (CIs) were used as measures of effect size. A P-valueof less than 0.05 (two-tailed) was considered to be statistically significant.

## Results

Most participants were from governmental health sectors (Table 1) and revealed an adequate level of knowledge regarding COVID-19. The sources of knowledge on the pandemic are varied, including current work institutions (94.5%), the WHO website (56%), scientific journals (42.7%), colleague healthcare (59.2%), and social media (41%). This explains the confusion mentioned by participants (62.3%) regarding the knowledge owing to rapidly changing recommendations and multi-sourced information. From the aspect of safety, 57.3% received relevant training on the use of PPE. The most frequently used PPE were surgical masks and gloves. The highly protective N95 masks were used by only 55.7%. Many healthcare workers (HCW; 68.3%) reported high levels of stress and anxiety about the COVID-19 pandemic.

**Table 1:** Participants’ characteristics and knowledge of COVID-19.

Parameter		Total (382) N (%)
Age		Mean 38.27±7.46
Gender	Male	213 (55.8)
	Female	169 (44.2)
Education	Board/doctorate degree or equivalent	97 (25.4)
	Master’s degree	161 (42.1)
	Bachelor’s degree	124 (32.5)
Years of experience	>20	68 (17.8)
	11-20	164 (43)
	6-10	69 (18)
	<5	81 (21.2)
Type of work facility	Governmental hospital clinic	84 (22)
	Private hospital clinic	78 (20.4)
	Governmental healthcare center	186 (48.7)
	Private healthcare center	34 (8.9)
Your source of information about COVID-19 (that all apply)	Current work institution	361 (94.5)
	WHO/CDC websites	214 (56)
	Scientific medical journals	163 (42.7)
	A colleague healthcare professional	226 (59.2)
	Social media	157 (41)
	Television	0 (0)
Do you feel confused regarding COVID-19 knowledge due to changing recommendations or multiple sources of information? (yes)		238 (62.3)
How do you rate the level of your knowledge on COVID-19?	Very poor	0 (0)
	Poor	13 (3.4)
	Good	296 (77.5)
	Very good	91 (19.1)
	Excellent	0 (0)
Have you been aware of measures of protection and risks for medical staff and patients? (yes)		368 (96.3)
Have you received relevant training on use of personal protective equipment? (yes)		219 (57.3)
Which one of the following PPEs do you use in your workplace? (that all apply)	Surgical masks	382 (100)
	Respirator N95 or FFP2 masks	213 (55.7)
	Face shields	86 (22.5)
	Gowns	218 (57)
	Gloves	376 (98.4)
	Protective glasses	31 (8.1)
How do you estimate your personal protection against COVID-19 during your medical practice (please choose from 1 = not protected to 5 = well protected)		(4)
Did you provide care for patients with suspected COVID-19? (yes)		162 (42.4)
Are you willing to provide care for suspected or confirmed COVID-19 positive patients? (yes)		219 (57.3)
During this COVID-19 pandemic, have you experienced any or more of the following symptoms: Fever, cough, runny nose, headaches, sore throat, and tiredness? (yes)		157 (41)
Do you isolate yourself (at home/another place) when you return back from your workplace daily? (yes)		24 (6.3)
Are you in agreement with your institution strategies to face the COVID-19 pandemic? (yes)		352 (92.1)
Do you feel stressed and anxious about this COVID-19 pandemic? (yes)		261 (68.3)

Canceled physical examination of the patients during the consultations was encountered most of the time (Table 2). 94.7% of participants experienced canceled patients' routine health check visits, 50.3% faced delays in consultation with other specialties, 84.3% had interruptions of chronic care visits, 55.5% encountered interruptions of acute care visits due to COVID-19, and 90.5% of participants sustained interruptions of preventive health visits. Also, most of the respondents noticed an increase in mental health visits. For patients with bronchial asthma, nebulizer use was modified (e.g., halted, shortened, or switched to home treatment) due to the COVID-19 pandemic by almost three-fourths of the respondents. Most of the study respondents think that COVID-19 measures (e.g., distancing, wearing face masks) adversely affect communication with patients. Almost two-thirds of participants reported the use of online consultations with their patients. Fortunately, there was no shortage of medications, nasopharyngeal swabs, or sanitizers. However, an occasional lack of PPEs occurred; almost half of the participants mentioned the shortage of HCWs due to COVID-19.

**Table 2:** Impact of COVID-19 on family medicine practices and treatment decisions.

Parameter			No (%)
Have you encountered a reduced number of outpatient visits?	No reduction		46 (12)
	< 25% reduction		58 (15.2)
	25-50% reduction		120 (31.4)
	50-75% reduction		158(41.4)
	>75% reduction		0 (0)
	Stopped all patient visits		0 (0)
Have you encountered a change in the consultation time of each outpatient visit?	No reduction		91 (23.8)
	< 25% reduction		206 (54)
	25-50% reduction		85 (22.2)
	50-75% reduction		0 (0)
	>75% reduction		0 (0)
Have you encountered canceled physical examinations of the patients during the consultation?	Not at all		0 (0)
	Sometimes		53 (13.9)
	Most of the time		201 (52.6)
	All the time		128 (33.5)
Do you think COVID-19 measures are adversely affecting communication with patients? e.g., distancing, wearing a face mask	Yes		246 (64.4)
	No		29 (7.6)
	May be		107 (28)
Have you encountered canceled routine health checks/elective procedures? (yes)			362 (94.7)
Have you encountered delays in consultation with other specialties? (yes)			192 (50.3)
Have you encountered a delay in the delivery of laboratory testing reports? (yes)			8 (2)
Have you encountered a delay in the delivery of the radiology testing report? (yes)			32 (8.4)
Have you experienced patient cancelation of health visits due to COVID-19, e.g., the panic of getting an infection? (yes)			342 (89.5)
Have you experienced the patient's inability to come for health visits due to COVID-19 quarantine measures and difficult transport? (yes)			184 (48.2)
Is the patient's COVID-19 condition (positive) impact treatment decisions? (yes)			382 (100)
Have you encountered interruption of chronic care visits due to COVID-19? e.g., difficult transportation or patient isolation (yes)			322 (84.3)
Have you encountered interruption of acute care visits due to COVID-19? (yes)			212 (55.5)
Have you encountered interruption of preventive health visits due to COVID-19? (yes)			346 (90.5)
Have you encountered an increase in mental health visits due to COVID-19 (yes)			287 (75.1)
Have you used telemedicine (online consultations) with your patients during the COVID-19 pandemic? (yes)			246 (64.4)
Is your practice considering implementing telemedicine visits, if COVID‐19 circumstances change in your region? (yes)			301 (78.8)
Has your practice modified inhalation nebulizer use due to the COVID‐19 pandemic for patients with bronchial asthma (e.g., halted, shortened, switched to home treatment, etc.)? (yes)			281 (73.5)
Impact on workforce			
Have you encountered a shortage of healthcare workers in family medicine clinics due to the COVID-19 pandemic?			242 (63.3)
Has your practice had a reduction in staff due to any of the following? (check all that apply)		Staff COVID‐19 illness	39 (10.2)
		Reduction in staffing due to reduced patient visits	61 (16)
		Staff transfer to other clinical areas/facilities	142 (37.2)
Has your practice experienced shortages or limited access to any of the following resources during the COVID‐19 pandemic? (check all that apply)		Chronic diseases medications	0 (0)
		Acute conditions medications	0 (0)
		Nasopharyngeal swabs for COVID‐19 specimen collection	0 (0)
		Medical hand sanitizer	0 (0)
		Personal protective equipment (e.g., N95 masks, surgical masks, other masks, gowns, gloves, etc.)	139 (36.4)
Have you encountered closure of your practice center due to COVID-19?			0 (0)
Are you routinely screening patients for COVID‐19 symptoms at the entrance to your office or in a space outside your office? (yes)			382 (100)
Is the infection control measures applied by your institution (e.g. triage before entry, and decontamination after each visit) delay patients’ care delivery?			0 (0)
Have you changed the physical arrangements in your office to enable staff and patients to follow physical distancing recommendations? (Check all that apply)	Alerted patients that they would NOT be able to bring a family member or friend to their appointment (unless circumstances require an exception)		83 (21.7)
	Requiring patients to wear masks while in the office		363 (95)
	Established triage stations outside the facility, clinic, or office to screen patients and visitors for COVID‐19 symptoms before they enter		382 (100)
	Installed barriers or social distancing mechanisms at front desks		374 (97.9)
	Converted or eliminated waiting area to allow for the distancing of at least six feet		352 (92.1)
	Reduced number of visits and/or increased time between visits		361 (94.5)
	Modified infusion suite to semi‐private space and/or use curtains as a barrier		271 (70.9)
Is your practice considering changing the scheduled visits of patients, if COVID‐19 circumstances change in your region? (yes)			331 (86.6)
Do you think this pandemic will have a lasting effect on the family medicine specialty? (yes)			316 (82.7)

## Discussion

This study was conducted during the COVID-19 outbreak and lockdown in Saudi Arabia. Family physicians here and around the world have to be up-to-date regarding the pandemic and how to protect themselves and their patients by following the proper precautions to prevent the spread of COVID-19. This pandemic has impacted clinical practice, knowledge, attitude, stress level, and workforce among family physicians since they encountered COVID-19 as frontline workers. According to our data, 41% of respondents considered social media to be a source of information, and a 2020 Pakistani study found that 78.68% of healthcare workers used social media as their primary source of information [18].

Only 57.3% of the family doctors have received relevant training on PPE use, including surgical masks, gloves, gowns, N95\Filtering Face Pieces 2 (FFP2) masks, face shields, and protective glass. However, all participants used a surgical mask and 98.4% used gloves. In comparison to a study done in Singapore published in 2020, all medical staff was trained on how to use the PPE appropriately. Furthermore, PPE use was mandatory for all patients at the counter, reception staff, and doctors during patients' consultation either in isolation or standard consultation rooms [19].

In this study, 36.4% of the participants reported a shortage of PPE access, and a Latin American study published in 2020 showed about 12% of healthcare providers had limited access to masks, and 11% had limited access to gloves. Another study published in 2020 among US and Pakistani doctors showed a similar lack of masks [20,21].

Almost half of our respondents (42.4%) have provided care for suspected or confirmed COVID-19 cases, and 57.3% are willing to provide care for suspected or confirmed cases of COVID-19. Moreover, 41% of the participants experienced fever, cough, runny nose, headache, and sore throat. In line with the previous studies, 24% of participants in an Italian study reported having at least one typical symptom of COVID-19 in the past 14 days, and 95% reported having been in close contact with positive COVID-19 patients [22]. Another study conducted in 2020 to determine the impact of the COVID-19 pandemic on residency training found that almost half of respondents (42.9%) had direct contact with COVID-19 patients; however, only 2.9% got an infection as a result of the working exposure [23].

Data obtained from our study suggest a reduction in outpatient visits according to study respondents; 41.4% estimated the reduction in visits to be 50-75%, and 31.4% estimated the reduction in outpatient visits to be around 25-50%. Half of our respondents (54%) have encountered a decrease in each outpatient visit's consultation time by less than 25%, while 23% of them reported no change in consultation time. Similar findings were reported in a study done by Verhoeven et al., in which 132 GPs were interviewed, and they reported that they see few people per day face-to-face, and only those with acute non-COVID problems, and that consultation and home visits are reduced to the minimum [24]. A study was done in the US that aimed to quantify changes in the volume and type of primary care delivered during the COVID-19 pandemic, and it showed that in 2020, the total number of office-based visits decreased by 50.2% in the second quarter (Q2) of 2020 compared with the second quarter of 2018-2019, while telemedicine visits increased from 1.1% of total Q2 2018-2019 visits to 35.3% in Q2 of 2020 [25]. Data from half of the physicians in our study indicate that they cancel physical examinations the majority of the time, and data from 33.5% indicate that they do not perform physical examinations at all. These results regarding visit-related problems may impact patient education about their medical conditions. Simultaneously, the restricted physical examination might lead to the patient's missing significant signs that would help diagnose the patient.

Most of the study respondents (64.4%) believe that COVID-19 measures affect communication with patients. Telemedicine was one of the options utilized by 64.4% of research participants in consultations to ensure communication with patients and check their health status. Some barriers to patient care using telephone consultations were reported in the study by Verhoeven et al., such as the loss of non-verbal communication, intercultural communication, and associated language problems [24]. Because less information can be obtained through telephone consultation, clinical decision-making is more complex and limited to patients' symptoms and self-examinations. Canceled routine health check/elective procedures were encountered by 94.7% of the study respondents. Half of them experienced a delay in consultation from other specialties.

According to all of the study respondents, the patient's COVID-19 condition (positive) impacts the treatment decisions. Data obtained from study respondents suggest increased interruption in preventive, chronic, and acute care visits because of COVID-19. Also, data suggest that 75.1% of the study population reported encountering an increase in mental health visits. On the other hand, 89.5% of study respondents encountered patients' cancellation of health visits due to fear of getting COVID-19, and 48.2% had experienced patients being unable to come because of the lockdown. On the other hand, there is no delay in laboratory tests or radiological imaging.

A shortage of healthcare workers was reported by 63.3% of the participants. According to the questionnaire, different situations are responsible for this reduction, such as reducing patient visits, staff being diagnosed with COVID-19, and transferring healthcare workers to other centers or facilities during the pandemic.

All participants routinely screen patients for COVID-19 symptoms outside or in the office since this symptoms-based screening is a useful tool for predicting COVID-19 cases. A previous study held in the US and published in 2020 agreed on the importance of this screening [26]. Most family physicians follow specific principles and guidelines mentioned by the World Health Organization to prevent the transmission of infection, such as instructing patients to wear face masks in the office, establishing triage stations outside the facility, installing barriers or social distance at the front desk, eliminating waiting areas to allow for more social space, and reducing the number of visits or increasing the time between the visits [27]. Only 21.7% of family physicians inform patients they would not bring a family member or friend unless there is an exception. These results are expected because of the government's plan to keep a distance to prevent COVID-19 spread.

This study has some limitations, including a lack of time and a small sample size, which limit the study's generalizability. An online survey was used to collect the data based on respondents' honesty and their recall ability. A longitudinal study design is an appropriate way to improve the effectiveness of the study.

## Conclusions

Pandemics of infectious diseases and the lockdown periods that follow them have a substantial adverse influence on family medicine practice. COVID-19 has reportedly interfered with preventive, chronic, and acute care visits and led to increased mental health visits. Additionally, outpatient visits have declined, and consultation time during each outpatient visit was reduced. Also, a shift from in-person clinics to telemedicine has occurred. These changes may perhaps lead to the possibility of diagnostic and medication refill delays.

## Appendices

Questionnaire to Assess the Impact of COVID-19 pandemic on family medicine practices in Saudi Arabia

Dear doctor, you are kindly invited to participate in this survey to assess the impact of the COVID-19 pandemic on family medicine and primary care practices in Saudi Arabia.

Information will be used for study purposes only, and will be kept confidential.

Time needed to respond to the survey items is approximately five minutes.

Your participation is highly appreciated.

1)    Participant’s demographics:

A)     Age:

B)     Gender:

1.       Male

2.      Female

C)     Education:

1.       Doctorate degree/Board degree

2.      Master’s degree

3.      Bachelor degree

D)     Position:

1.       Consultant/Professor/Assistant professor

2.       Specialist/Registrar

3.       Resident/general practitioner

E)     Years of experience:

1.       0- 5 years

2.      6-10 years

3.      11-20 years

4.      More than 20 years

F)     Sector of activity:

1.       Governmental hospital clinic

2.       Private hospital clinic

3.       Governmental healthcare center

4.       Private healthcare center

2)    COVID-19 Knowledge and prevention of infection

A)     Have you received specific training or recommendations about COVID-19 from your institution

1.       Yes

2.       No

B)     Your source of information about COVID-19:

1.       Your institution (hospital, clinic)

2.      WHO/CDC websites

3.      Scientific medical journals

4.      Colleague healthcare professionals

5.      Social media

6.      TV and Media

C)     Do you feel well informed on the latest COVID-19 guidelines?

1.       Yes

2.       No

D)     Do you feel confusion regarding COVID-19 knowledge due to changing recommendations or multiple sources of information?

1.      Yes

2.      No

E)     How do you rate the level of your knowledge on COVID-19?

1.       Very well informed

2.      Well informed

3.      Somewhat informed

4.      Poorly informed

F)     Have you been aware about measures of protection and risks for medical staff and patients?

1.       Yes

2.       No

G)    Have you received relevant training on use of personal protective equipment?

1.       Yes

2.       No

H)    Which one of the following PPEs do you use in your workplace?

1.       Medical masks

2.      Respirator N95 or FFP2 masks

3.      Face shields

4.      Gloves

5.      Gowns

6.      Protective glasses

7.      None

I)       How do you estimate your personal protection against COVID-19 during your medical practice [Please choose from 1 = not protected to 5 = well protected]?

1.                 2.             3.                   4.                      5.

J)      Did you provide care for patients with positive COVID-19?

1.       Yes

2.       No

K)    Are you willing to provide care for suspected or confirmed COVID-19 positive patients?

1.       Yes

2.       No

L)     During this COVID-19 pandemic, have you experienced any or more of the following symptoms: Fever, cough, runny nose, headaches, sore throat, tiredness?

1.       Yes

2.       No

M)   What would be your action if you suspect COVID-19 in yourself?

1.       Self-isolation at home until symptoms improve

2.      Call a specific COVID-19 phone number

3.      Continue to provide patient care wearing a face mask

4.      Continue practice as usual until case confirmed/excluded

N)     Do you isolate yourself (at home/other place) when you return back from your work place?

1.       Yes

2.       No

O)    Do you feel stressed and anxious about this COVID-19pandemic?

1.       Yes

2.       No

3) Impact on family medicine practices and treatment decisions:

Are you in agreement with your institution strategies to face the COVID-19 pandemic?

1.       Yes

2.       No

3.       Not sure

Have you encountered reduced number of outpatient visits?

1.      No reduction (as usual)

2.      <25% reduction

3.      25-50% reduction

4.      50-75% reduction

5.      75% reduction

6.       Stopped all outpatient visits

Have you encountered change in the consultation time of each outpatient visits?

1.         No change (same time as usual)

2.        <25% reduction

3.         25-50% reduction

4.         50-75% reduction

5.         75% reduction

Have you encountered canceled physical examinations of the patients during the consultation?

1.       Not at all

2.       Sometimes

3.       Most of the time

4.       All the time

Do you think COVID-19 measures are adversely affecting communication with patients, e.g., distancing, wearing a face mask?

1.       Yes

2.       No

3.       Maybe

Have you encountered canceled preventive/routine health checks/elective procedures?

1.       Yes

2.       No

Have you encountered interruption of chronic care visits due to COVID-19, e.g., difficult transportation or patient isolation?

1.      Yes

2.      No

Have you encountered interruption of acute care visits due to COVID-19?

1.       Yes

2.       No

Have you encountered delays in consultation with other specialties?

1.       Yes

2.       No

Have you encountered a delay in the delivery of laboratory testing reports?

1.       Yes

2.       No

Have you encountered delay in delivery of laboratory report?

1.       Yes

2.       No

Have you encountered delay in delivery of radiology testing report?

1.       Yes

2.       No

Have you experienced patient cancellation of health visits due to COVID-19, e.g., the panic of the negative effect on immunity?

1.       Yes

2.       No

Have you experienced patient cancellation of treatment due to COVID-19, e.g., the panic of the negative effect on immunity?

1.       Yes

2.       No

Have you experienced patient inability to come for health visits due to COVID-19, e.g., quarantine measures and difficult transport?

1.       Yes

2.       No

Have you encountered increase in mental health visits due to COVID-19?

1.       Yes

2.       No

Is patient COVID-19 condition (positive) impact treatment decisions?

1.       Yes

2.       No

3.       Occasionally

Have you encountered treatment interruption due to patient isolation?

1.       Yes

2.       No

Have you used telemedicine (online consultations) with your patients during the COVID-19 pandemic?

1.       Yes

2.       No

Is your practice considering implementing telemedicine visits, if COVID‐19 circumstances change in your region?

1.       Yes

2.       No

Has your practice modified inhalation nebulizer use due to the COVID‐19 pandemic for patients with bronchial asthma (e.g., halted, shortened, switched to home treatment, etc.)?

1.       Yes

2.       No

Has your practice modified intravenous drug infusions due to the COVID‐19 pandemic (e.g., halted, shortened, switched to oral, etc.)?

1.       Yes

2.       No

4)   Impact on Workforce:

Have you encountered shortage of healthcare workers in family medicine/primary care clinics due to COVID-19 pandemic?

1.       Yes

2.       No

Has your practice had a reduction in staff due to any of the following? (check all that apply)

1.       Staff COVID‐19 illness

2.      Reduction in staffing due to reduced patient visits

3.      Staff transfer to other clinical areas/facilities

Have you encountered reduced supply of medications/equipment by your institution?

1.       Yes

2.       No

Has your practice experienced shortages or limited access to any of the following resources during the COVID‐19 pandemic? (check all that apply)

1.        Chronic diseases medications

2.       Acute conditions medications

3.       Nasopharyngeal swabs for COVID‐19 specimen collection

4.       Medical hand sanitizer

5.       Personal protective equipment (e.g., N95 masks, surgical masks, other masks, gowns, gloves, etc.)

6.       Other

7.       No, our practice is not experiencing any of the above shortages.

Are you routinely screening patients for COVID‐19 symptoms at the entrance to your office or in a space outside your office?

1.       Yes

2.       No

Are the infection control measures applied by your institution (e.g., triage before entry and decontamination after each visit) delays patients’ care delivery?

1.       Yes

2.       No

Have you changed the physical arrangements in your office to enable staff and patients to follow physical distancing recommendations? (check all that apply)

1.       Alerted patients that they would not be able to bring a family member or friend to their appointment (unless circumstances require an exception)

2.       Requiring patients to wear masks while in the office

3.       Established triage stations outside the facility, clinic, or office to screen patients and visitors for COVID‐19 symptoms before they enter

4.       Installed barriers or social distancing mechanisms at front desks

5.       Converted or eliminated waiting area to allow for the distancing of at least six feet

6.       Reduced number of visits and/or increased time between visits

7.       Modified infusion suite to semi‐private space and/or use curtains as a barrier

8.       Suspended or moved to a virtual platform, all on‐site group and patient wellness and support activities (e.g., yoga, education seminars, support groups, etc.)

9.       Other

10.     We have made no changes to the physical arrangements

Is your practice considering changing the scheduled visits of patients not on active treatment, if COVID‐19 circumstances change in your region?

1.       Yes

2.       No

Have you had your salary reduced during the pandemic lockdown?

1.      Not at all

2.     <25% reduction

3.      25-50% reduction

4.      50-75% reduction

5.      >75% reduction

Do you think this pandemic will have a lasting effect on the family medicine specialty?

1.      Yes

2.      No

THANKS
